# Somatic copy number gains in *MYC, BCL2*, and *BCL6* identifies a subset of aggressive alternative-DH/TH DLBCL patients

**DOI:** 10.1038/s41408-020-00382-3

**Published:** 2020-11-09

**Authors:** Jordan E. Krull, Kerstin Wenzl, Keenan T. Hartert, Michelle K. Manske, Vivekananda Sarangi, Matthew J. Maurer, Melissa C. Larson, Grzegorz S. Nowakowski, Stephen M. Ansell, Ellen McPhail, Thomas M. Habermann, Brian K. Link, Rebecca L. King, James R. Cerhan, Anne J. Novak

**Affiliations:** 1 Mayo Clinic Graduate Program in Biomedical Science, Mayo Clinic, Rochester, MN USA; 2 Division of Hematology, Mayo Clinic, Rochester, MN USA; 3 Department of Health Sciences Research, Mayo Clinic, Rochester, MN USA; 4 Division of Hematopathology, Mayo Clinic, Rochester, MN USA; 5 Division of Hematology, University of Iowa Hospitals and Clinics, Iowa City, IA USA

**Keywords:** Cytogenetics, Oncogenes, B-cell lymphoma

## Abstract

Double/triple hit lymphoma (DH/TH), known as high-grade B-cell lymphoma (HGBL), is an aggressive diffuse large B cell lymphoma (DLBCL), defined as having concurrent *MYC*, *BCL2*, and/or *BCL6* gene rearrangements. While gene rearrangements represent significant genetic events in cancer, copy number alterations (CNAs) also play an important role, and their contributions to rearrangements have yet to be fully elucidated. Using FISH and high-resolution CNA data, we defined the landscape of concurrent gene rearrangements and copy gains in *MYC*, *BCL2*, and *BCL6*, in a cohort of 479 newly diagnosed DLBCL. We also show that concurrent translocations and copy number alterations, in combinations similar to DH/TH, identify a unique subset of DLBCL, alternative DH/TH, that have survival outcomes similar to DH/TH DLBCL patients.

## Introduction

Diffuse large B-cell lymphoma (DLBCL) is the most common aggressive non-Hodgkin lymphoma subtype in Western countries^1,2^. Factors that predict DLBCL prognosis include clinical characteristics^3^, cell-of-origin (COO)^4,5^, *MYC* translocation status^6–8^, and genomic alterations^9–11^. Concurrent rearrangements of *c-MYC* and *BCL2* and/or *BCL6* detected by fluorescence in situ hybridization (FISH), also known as “double hits” (DH), occur with a frequency of 6–14%^6,12^, are associated with transcriptional dysregulation of *MYC*, and have been correlated with poor prognosis in patients treated with chemotherapy alone or with the addition of rituximab^6,12–14^. In the revised fourth edition of the World Health Organization (WHO) classification of lymphoid neoplasms published in 2016^15^, DH/triple-hit (TH) DLBCL was reclassified as high-grade B-cell lymphoma, with *MYC* and *BCL2* and/or *BCL6* rearrangements. In addition, double-expressor lymphoma, defined as overexpression of MYC and BCL2 proteins without chromosomal rearrangements, although not a distinct WHO classification, accounts for 20–30% of DLBCL cases and also has poor outcomes^13^.

While translocations in *MYC*, *BCL2*, and *BCL6* represent significant genomic events in lymphoma, less is known about the contribution of chromosomal copy number alterations (CNAs) in these genes to existing translocations. *MYC* copy gains are detectable in DLBCL and have been shown to increase *MYC* mRNA expression in DLBCL^16–19^. Likewise, *MYC* translocation negative patients have been shown to have MYC IHC scoring similar to those harboring the translocation, alluding to alternative mechanisms driving *MYC* expression in DLBCL^13,20^. While the DH/TH cases have concurrent rearrangement events in *MYC*, *BCL2*, and *BCL6*, it is not clear whether an expanded definition could include copy number gains as a secondary event. To date, two studies have examined the impact of both translocations and CNAs, occurring *in* *trans*, in *MYC*, *BCL2*, and/or *BCL6*, detected by FISH. Quesada et al. and Li et al. both identified atypical DH/TH cases in large cohorts, with concurrent *MYC* and *BCL2* and/or *BCL6* abnormalities, other than double translocation, which had outcomes similar to bona fide DH/TH. However, several limitations exist in regards to the analysis within these studies, including limited definition, *BCL6* exclusion, case selection, and/or copy number detection method^18,21^. Lu et al.^22^ similarly suggested that *MYC* and *BCL2* double CNA (*in cis)* constituted a group of high-risk DLBCL, though *MYC and BCL2* were never analyzed in combination with FISH, as in the previous studies. Likewise, two of the aforementioned studies acknowledge *BCL6* as a defining gene for DH/TH lymphoma, yet neither included it in their analysis. Understandably, it is very difficult to acquire data on enough patients to power investigations into relatively rare subtypes, such as these. These studies, in addition to many others, rely on the existing clinical FISH data to roughly determine gene-specific CNAs. High-resolution copy number analyses, such as copy number arrays, are not routinely performed on DLBCL patients and are not commonly available. Therefore, it remains to be defined whether or not translocations and copy number events in *MYC*, *BCL2*, and *BCL6* cooperate to identify new DH-like or atypical-DH lymphoma groups. In addition, the relative prevalence of these cases in the DLBCL population and the clinical outcome of these cases is yet to be fully explored. In this study, we define the landscape of *MYC*, *BCL2*, and *BCL6* translocations in combination with CNAs in two independent cohorts of DLBCL and address the key question of how CNAs impact DLBCL prognosis.

## Methods

### Study populations

For this study, newly diagnosed and consented DLBCL patients (*n* = 260) were recruited and prospectively followed through the Molecular Epidemiology Resource (MER) of the University of Iowa/Mayo Clinic Lymphoma Specialized Program of Research Excellence. Full details of this prospective cohort study of lymphoma outcomes have been previously published^23^. This study was approved by the Mayo Clinic Institutional Review Board. This analysis was restricted to DLBCL patients treated with frontline immunochemotherapy and who had available FISH and CNV data. COO on available MER cases was determined by gene expression profiling (*n* = 15)^24^, Lymph2Cx assay (NanoString, *n* = 106)^25^, or the Hans algorithm (*n* = 110)^26^. An additional 219 DLBCL cases were included from Chapuy et al.^9^ and were acquired from the journal website Supplementary Data Tables 2 and 8. Some patients from this study overlapped with patients in the Mayo cohort and were removed from the analysis. In all, 219 unique samples from this study had whole-exome sequencing, which was used to detect structural variants and CNAs bioinformatically. Baseline clinical characteristics of the Mayo and Chapuy et al. cases included in this study are shown in Supplemental Table 1.

### FISH analysis

For the Mayo Cohort, translocation events in *MYC* (8q24.1), *BCL2* (18q21), and *BCL6* (3q27) were determined by FISH break-apart probes (Abbott Laboratories, Des Plaines, IL, USA) on tissue microarray (TMA) slides, according to standard protocols. Briefly, TMAs were deparaffinized, renatured, and dehydrated. Probe working solutions were mixed using 1 μL of the concentrated probe (Abbott Laboratories, Des Plaines, IL, USA) with 9 μL of LSI/WCP^®^ hybridization buffer (Abbott Laboratories). The working solutions of BAP BCL6(3q27), BAP MYC(8q24.1), and BCL2(18q21) were applied to the target areas, coverslipped, co-denatured with a ThermoBrite^™^ at 83 °C for 5 min, and hybridized overnight in a 37 °C humidified oven. Following hybridization, slides were soaked in room temperature (RT) 2×SSC/0.1% NP-40 to remove coverslips, placed in 2×SSC/0.1% NP-40 at 74 °C for 2 min and then placed into RT 2×SSC/0.1% NP-40 for 2 min. The slides were stained with 4′-6,-diamidino-2-phenylindole (Vector Laboratories) and coverslipped. The slides were analyzed by a technologist using standard fluorescence microscopy methods. Tissue samples were scanned in their entirety, and the qualitative result was determined based on observed signal patterns. Visualization of the FISH signals was accomplished by using the fluorescence microscope, and pictures were captured by using a FISH imaging system (CytoVision, Leica Biosystems).

### Copy number analysis

Methods for analysis of copy number, including sample collection, analysis, and data interpretation has been previously published^27^. Briefly, DNA, extracted from formalin-fixed, paraffin-embedded (FFPE) DLBCL tumors (*n* = 247), was processed at the Mayo Clinic Cytogenetics Lab using the molecular inversion probe OncoScan^TM^ FFPE Assay Kit (Affymetrix, Santa Clara, CA, USA). For an additional 13 cases, whole-exome sequencing files were used. Raw WES bam files and OncoScan OSCHP files were analyzed using Nexus Copy Number 9.0 software (Biodiscovery, El Segundo, USA). Data interpretation and copy number calling were made using the human reference genome GRCh37/hg19. Nexus standard configuration for gain calling thresholds was used. All gains and high-copy-gains (amplification) are reported in this study as copy number gains. Copy number losses were excluded from this analysis.

### Extended cohort translocation and copy number

Gene-specific structural variants and copy number gains were obtained from Supplementary Table 8, gene sample matrix, from Chapuy et al.^9^. Presence or absence of *MYC*, *BCL2*, and *BCL6* structural variants was reported from sequencing data as 0 (Absence), and 1 or 3 (presence). Copy number gains were reported as 0 (Absent), and 1 or 2 (Present) for *MYC* (8q24.22), *BCL2* (18q/18q21.33), and *BCL6* (3q/3q28). Both arm length and q-band gains/amplifications were considered for *BCL2* and *BCL6*.

### MYC, BCL2, and BCL6 protein expression

Positive protein expression was assayed by immunohistochemistry in the Mayo Clinic Pathology Research Core using standard MYC, BCL2, and BCL6 clinical antibodies. Positivity was determined according to published methods: MYC-IHC+ (≥40% of cells), BCL2-IHC+ (≥50% of cells), and BCL6-IHC+ (≥30% of cells). Protein expression was completed in 192 samples for MYC, 201 samples for BCL2, and 41 samples for BCL6. Scoring was completed by a hematopathologist in the Mayo Clinic Department of Lab Medicine and Pathology.

### Statistical analysis

Chi-square test-of-independence was performed using GraphPad Prism version 8.1.1 for Windows, GraphPad Software, La Jolla, CA, USA, www.graphpad.com. Survival data were analyzed using a cox-proportional-hazards model within the Survminer package in R^28,29^. Sensitivity analysis was used to adjust the model for single covariates, one at a time, within key clinical subsets of patients within the cohort. All hazard ratios (HR), 95% confidence intervals, and Kaplan–Meier estimates were generated from the package.

## Results

### Copy gains in *MYC*, *BCL2*, and *BCL6*, in addition to translocations, contribute to the diversity of DLBCL DH subsets

**Fig. 1:**
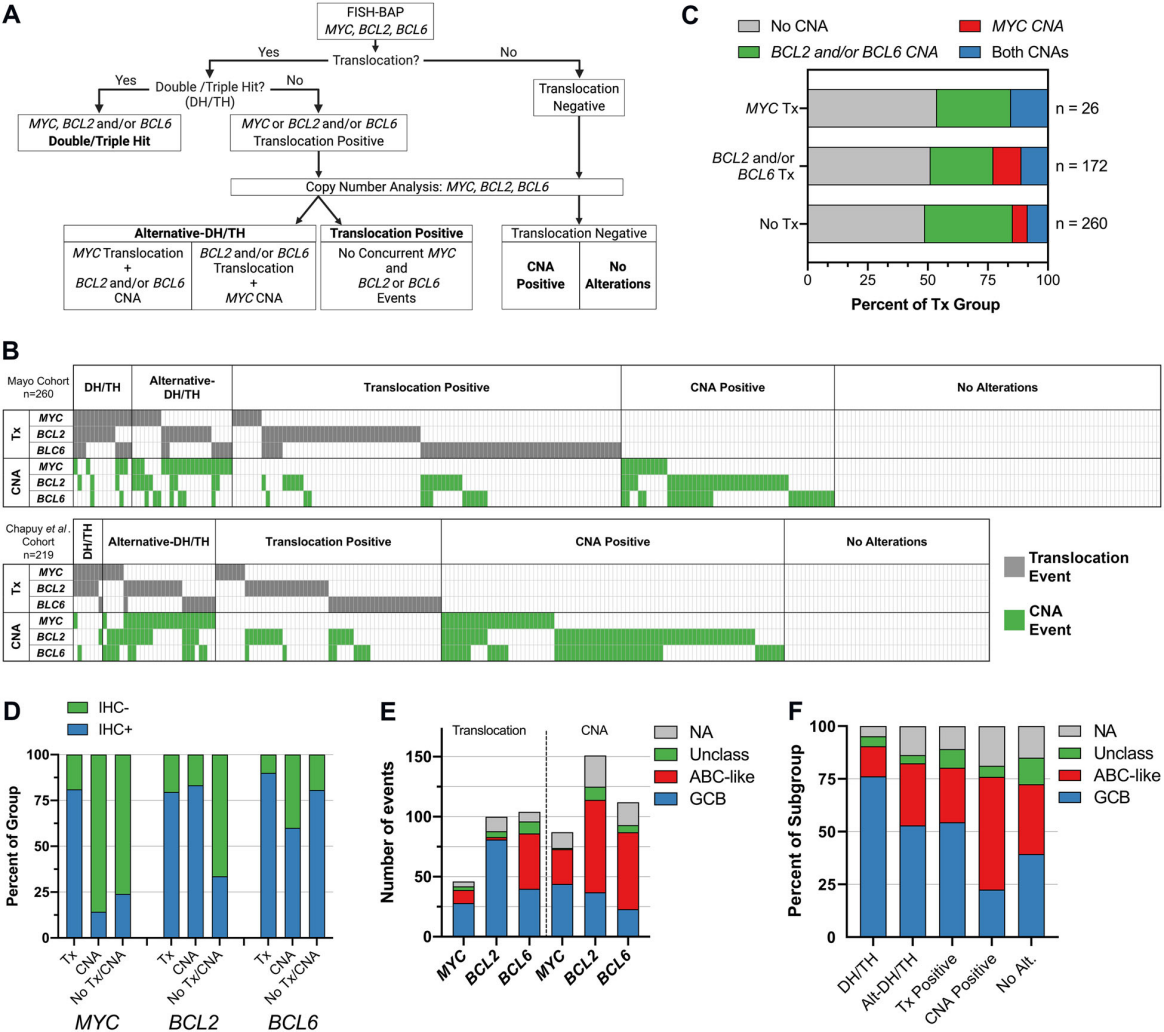
Identification and prevalence of *MYC*, *BCL2*, and *BCL6* translocations and copy number gains. **A** Analysis workflow for identification of *MYC*, *BCL2*, and *BCL6* translocation and/or can subgroups. **B** The landscape of translocations and CNAs (only copy number gains are shown), among DH/TH, Alt-DH/TH, translocation positive, copy gain positive, and no alteration subgroups in the Mayo Cohort (upper panel, *n* = 260) and the Chapuy et al. Cohort (lower panel, *n* = 219). **C** Frequencies of CNA combinations in translocation subgroups, excluding DH/TH. **D** Percentage of patients positive and negative for protein expression (MYC *n* = 192, positive ≥ 40% cells; BCL2 *n* = 201, positive ≥ 50% cells; BCL6 *n* = 41, positive ≥ 30% cells) according to their translocation (Tx) and CNA status. **E** Total number of Translocation or CNA events for each alteration by COO. **F** Percentage of each COO subgroup according to their genetic subgroup.

In order to define the landscape and ascertain the impact of concurrent translocations and copy number gains in *MYC* and *BCL2* and/or *BCL6*, FISH and copy number data from 260 DLBCL patients, from the Mayo/Iowa MER (Mayo Cohort), were analyzed. The analysis flow for the study is shown in Fig. 1A. Translocation events were first evaluated by FISH to identify patients with translocations in *MYC*, *BCL2*, or *BCL6*. This identified three groups: DH/TH cases; those with one or more translocations, but not defined as DH/TH (translocation positive, Tx positive); and those with no translocations (translocation negative). We next evaluated copy number gains, henceforth referred to as CNA, in *MYC*, *BCL2*, and *BCL6* in the same patients using OncoScan Array. In this analysis, translocation-negative cases were broken into those with copy number gains (CNA positive) and those without translocations or copy number gains (no alteration). From the translocation-positive cohort, we identified a subset of patients that had concurrent events in *MYC* and *BCL2* and/or *BCL6*, but a translocation in one gene and a CNA in another, distinguishing a subset of a non-canonically defined DH/TH (alternative-DH/TH, Alt-DH/TH). The results from this analysis highlight a complex landscape of translocation and CNAs (Fig. 1B, upper panel). Using the same classification workflow, an additional, 219 unique DLBCL patients were analyzed from a publicly available DLBCL dataset (Chapuy et al.^9^), in which structural variants and copy number gains in *MYC* and *BCL2* and/or *BCL6* were identified (Fig. 1B, lower panel). The combined analysis (*n* = 479) identified 21 (4.4%) DH/TH cases, 51 (10.6%) Alt-DH/TH cases, 147 (30.7%) Tx positive cases, 133 (27.8%) CNA positive cases, and 127 (26.5%) with no alterations. A summary of the number of cases in each subgroup and the clinical characteristics of each cohort is shown in Supplemental Table 1. When excluding DH/TH cases, we found that copy number data provided additional information to the FISH results in regards to the genomic status of each gene. Nearly 50% of positive translocation cases had copy gains in one or more of the three genes and were considered Alt-DH/TH (Fig. 1C). Of potential significance, nearly half of all *MYC* translocations have concurrent *BCL2* and/or *BCL6* gains (Fig. 1C). In addition, nearly 25% of patients with *BCL2* and/or *BCL6* translocation have a concurrent *MYC* gain.

The pathogenic mechanism resulting from translocations in *MYC*, *BCL2*, and *BCL6*, is protein overexpression and deregulation of cell proliferation and apoptosis. The impact of CNAs on protein expression in DLBCL is less clear. To assess the effect of CNAs on expression, we obtained immunohistochemistry data on available Mayo Cohort cases (*N* = 221). MYC, BCL2, and BCL6 positivity were defined as an expression by ≥40%, ≥50%, and ≥30% of malignant cells, respectively, in accordance with established cutoffs^7,13^. Positive MYC expression was detected in 81.0% of *MYC* translocation cases and 14.3% of *MYC* CNA cases, suggesting that only a small subset of *MYC* CNAs result in elevated protein expression (Fig. 1A). In contrast, positive BCL2 and BCL6 expression were detected at similar levels in both the translocation (79% and 90%) and CNA cases (83% and 60%), supporting the idea that elevated BCL2 and BCL6 expression can be driven by multiple mechanisms, including CNA (Fig. 1D).

DH/TH lymphomas are predominantly derived from germinal center B (GCB) cell COO subtypes typically resulting from *BCL2* translocations^13,30^. Similarly, when we analyzed all cases positive for any translocations (*n* = 219), a majority (56%) were found in GCB cases. However, the addition of copy number data yields a much different story, where activated B cell-like (ABC) cases were found to be enriched for copy number gains in *MYC*, *BCL2*, and *BCL6* (Fig. 1E). Given this, we hypothesized that an Alt-DH/TH cases might include more ABC-like DLBCL. Indeed, compared to DH/TH (14.2% ABC), Alt-DH/TH were enriched for ABC cases (29.4%). Together, this data further expands our understanding of the *MYC*, *BCL2*, and *BCL6* genomic landscape highlights the contribution of CNA data and identifies a unique Alt-DH/TH population in which ABC-DLBCL is more prevalent when compared to DH/TH.

### Clinical features and outcome of Alt-DH/TH patients

While the detrimental prognostic impact of having DH/TH DLBCL has been well-documented^31^, little is known about the contribution of CNAs, in particular, the impact on patient outcomes. Therefore, we compared the clinical characteristics and outcome of Alt-DH/TH cases with DH/TH and all other groups combined. The survival curves for translocation-positive, copy-gain-positive, and no alterations, and event combinations within these groups, were overlapping and statistically indistinguishable; we, therefore, combined them and considered this combined group as controls (Other) for statistical calculations (Supplementary Fig. 1). Compared to the DH/TH and Other DLBCL cases, Alt-DH/TH patients were clinically similar. There were no significant differences within baseline clinical measures between Alt-DH/TH and Other DLBCL (Table 1). As expected, the DH/TH cases exhibited a significant enrichment for GCB COO (Table 1).

**Table 1:** Clinical characteristics by group.

Measurement	Other (Control)	DH/TH	*p*	Alt-DH/TH	*p*
*N*	407 (85.0%)	21 (4.4%)		51 (10.6%)	
*Age*			0.56		0.36
>60	286 (70.3%)	16 (76.2%)		39 (76.4%)	
≦60	121 (29.7%)	5 (23.2%)		12 (23.6%)	
*Sex*			0.14		0.96
Male	230 (56.5%)	11 (52.4%)		29 (56.9%)	
Female	177 (43.5%)	10 (47.6%)		22 (43.1%)	
*IPI*			0.50		0.31
0–1	160 (39.3%)	5 (23.8%)		19 (37.3%)	
2	99 (24.3%)	6 (28.6%)		10 (19.6%)	
3	97 (23.8%)	6 (28.6%)		18 (35.3%)	
4–5	49 (12.0%)	4 (19.0%)		4 (7.8%)	
	NA = 2				
*Stage*			0.13		0.96
I–II	185 (45.5%)	6 (28.6%)		23 (45.1%)	
III–IV	222 (54.5%)	15 (71.4%)		28 (54.9%)	
*COO*			0.01		0.24
ABC-like	151 (37.1%)	3 (14.2%)		15 (29.4%)	
GCB	160 (39.3%)	16 (76.2%)		27 (52.9%)	
Unclassified	36 (8.8%)	1 (4.8%)		2 (3.9%)	
NA	60 (14.7%)	1 (4.8%)		7 (13.8%)	
*Cohort*			0.27		0.31
Mayo	222 (54.6%)	14 (66.7%)		24 (47.1%)	
Chapuy et al.	185 (45.4%)	7 (33.3%)		27 (52.9%)	

*p* chi-square *p* value, *IPI* international prognostic index, *COO* cell-of-origin, *ABC*-like activated B-cell like, *GCB* germinal center B-cell, *NA* not applicable.

**Fig. 2:**
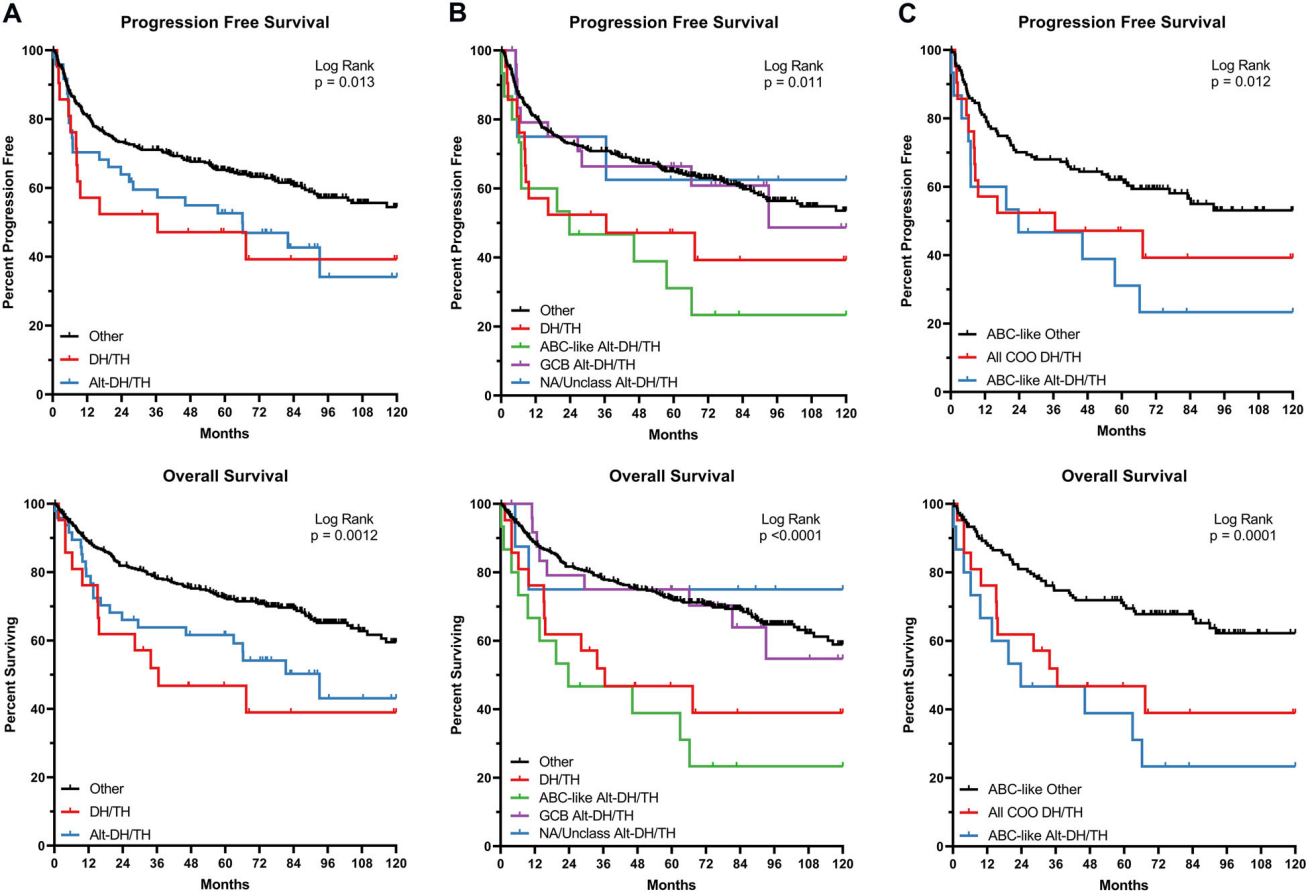
Survival outcomes of Alt-DH/TH patients. Kaplan–Meier survival curves for event-free/progression-free (EFS/PFS) and overall survival (OS) of DH/TH, Alt-DH/TH, and Other (translocation positive + copy gain positive + no alteration patients). Progression-free survival is a combination of EFS from one study and PFS from another. **A** Progression-free survival (*p* = 0.013) and overall survival (*p* = 0.0012) of all Alt-DH/TH (*n* = 51), DH/TH (*n* = 21), and Other cases (*n* = 410) in the combined cohort. **B** Progression-free survival (EFS/PFS) (*p* = 0.011) and overall survival (*p* < 0.001) of Alt-DH/TH cases separated by COO class (ABC-like, *n* = 15; GCB, *n* = 25; NA/unclassifiable, *n* = 8), all DH/TH (*n* = 21), and all Other cases (*n* = 410). **C** Progression-free survival (EFS/PFS) (*p* = 0.012) and overall survival (*p* = 0.001) of ABC-like Alt-DH/TH (*n* = 15), DH/TH (*n* = 21), and ABC-like Other (*n* = 151) cases in the combined cohort. *p* Values listed on each graph were derived from a Log Rank test.

To determine if Alt-DH/TH have similarly poor outcomes to DH/TH patients, we compared the event-free/progression-free survival (EFS/PFS) and OS of all three groups. The Kaplan–Meier curves for EFS/PFS (upper panel) and OS (lower panel), between DH/TH, Alt-DH/TH and Other, are significantly different (Fig. 2A, Log Rank, *p* = 0.013 and *p* = 0.0012, respectively). Because of the increased prevalence of ABC cases in the Alt-DH group, compared to DH/TH, we performed a secondary analysis based on COO (Fig. 2B). The Alt-DH/TH patients, which had an ABC-like COO, appeared to be the primary contributor to the poor survival outcomes of Alt-DH/TH patients, compared to Other patients of all COOs and had nearly overlapping EFS/PFS and OS curves with canonical DH/TH. In order to determine the effect of COO in controls, ABC-like Other cases were plotted along with ABC-like Alt-DH/TH and all DH/TH, which showed the same trends (Fig. 2C). Results from Kaplan–Meier estimates of progression at 24-months (EFS24) and 5-year OS in these subsets are listed in Table 2.

**Table 2:**
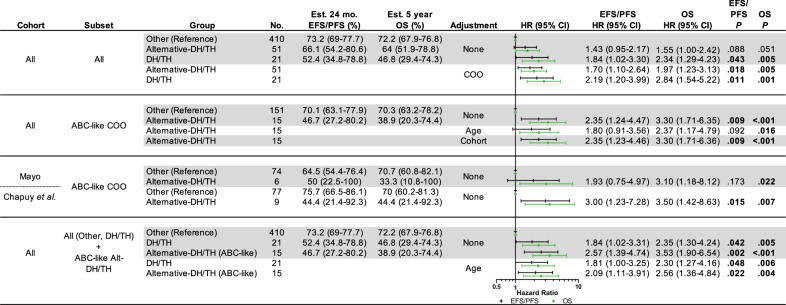
Survival outcomes and hazard models of alternative-DH/TH for event/progression free and overall survival.

*Est.* estimated, *mo.* months, *EFS/PFS* event free survival/progression free survival, *OS* overall survival, *HR* hazard ratio, *COO* cell-of-origin, *ABC-like* activated B cell-like, *Alt-DH/TH* alternative-DH/TH.

In order to determine group-specific effect sizes and significance, EFS/PFS and OS were modeled using a cox proportional hazards regression analysis (Cox-PH model). Using Other DLBCL (*n* = 410) as reference, we observed significant HRs for EFS/PFS in the DH/TH group and HRs suggestive of poor OS in both DH/TH and Alt-DH/TH (*p* = 0.005, *p* = 0.051, respectively). Consistent with our previous result, ABC-like Alt-DH/TH (*n* = 15) cases had significantly poor HRs for EFS/PFS and OS (*p* = 0.009 and *p* < 0.001, respectively), when compared to ABC-like Other cases (*n* = 151) as reference. The outcomes were consistent within each cohort and remained consistent when adjusting for age and cohort, independently, in a multivariate model (Table 2). Finally, ABC-Alt-DH/TH and all DH/TH cases had significantly poor HRs for EFS/PFS and OS, which were relatively overlapping and were unaffected when adjusting for age (Table 2).

## Discussion

We have described the landscape of concurrent copy number gains and translocations in *MYC*, *BCL2*, and *BCL6* in a large cohort of DLBCL patients combined from two independent studies. Our study identifies a unique set of cases that have concurrent translocations and copy number gains in *MYC*, *BCL2*, and/or *BCL6* outside of DH/TH lymphoma that cannot readily be identified using standard FISH alone. More importantly, our study suggests that Alt-DH/TH patients have outcomes similar to DH/TH cases, which may be particularly associated with an ABC-like COO. Our work aligns with recent studies defining a double hit gene signature^20,32^ and may explain why as many as 30% of patients with DLBCL have concurrent overexpression of *MYC* and *BCL2* mRNA or protein (double expression) independent of DH/TH translocation status. Our work also corroborates prior studies, as many previous definitions of Alt-DH/TH share similar features and outcomes with this study^18,21^. However, this study includes an expanded definition, with *BCL6* translocations and copy number events, and utilized high-resolution copy number analysis, which was not included in prior studies. Interestingly, *MYC* copy gains were not correlated with positive expression by IHC, unlike *BCL2* and *BCL6*. Despite this, negative outcomes were seen in Alt-DH/TH patients of ABC-like COO, which includes most of the *BCL6-*Tx*/MYC-*CNA cases. Furthermore, our work complements recent results from Hilton et al.^33^ that identify double hit gene signature positive patients with alternative genomic events, cryptic to standard FISH, affecting *MYC*, *BCL2*, and downstream pathways.

The proportion of Alt-DH/TH within all ABC-like cases in this study (8.8%) was similar to the proportion of DH/TH within all GCB cases in this study (7.8%) and therefore could be important for future screening approaches. Ongoing studies may further connect additional somatic events which correlate with the ABC-like Alt-DH/TH and further explain the poor clinical outcome of these patients. However, based on our existing results, we can conclude that Alt-DH/TH patients with ABC-like COO are a particularly aggressive subgroup of DLBCL and have outcomes resembling that of canonical DH/TH patients. Moving forward, it will be important to independently validate our findings on the impact of Alt-DH/TH on DLBCL outcome and consider assessing CNAs clinically.

## Supplementary information

Supplemental Figures

## References

[1] Howlader, N. et al. *SEER Cancer Statistics Review, 1975–2010.* https://seer.cancer.gov/archive/csr/1975_2010/ (National Cancer Institute. Bethesda, MD, November 2012 SEER data submission, posted to the SEER web site, April 2013).

[2] Sant M (2010). Incidence of hematologic malignancies in Europe by morphologic subtype: results of the HAEMACARE project. Blood.

[3] A predictive model for aggressive non-Hodgkin’s lymphoma. (1993). The International Non-Hodgkin’s Lymphoma Prognostic Factors Project. N. Engl. J. Med..

[4] Alizadeh AA (2000). Distinct types of diffuse large B-cell lymphoma identified by gene expression profiling. Nature..

[5] Wright G (2003). A gene expression-based method to diagnose clinically distinct subgroups of diffuse large B cell lymphoma. Proc. Natl Acad. Sci. USA.

[6] Barrans S (2010). Rearrangement of MYC is associated with poor prognosis in patients with diffuse large B-cell lymphoma treated in the era of rituximab. J. Clin. Oncol..

[7] Savage KJ (2009). MYC gene rearrangements are associated with a poor prognosis in diffuse large B-cell lymphoma patients treated with R-CHOP chemotherapy. Blood.

[8] Klapper W (2008). Structural aberrations affecting the MYC locus indicate a poor prognosis independent of clinical risk factors in diffuse large B-cell lymphomas treated within randomized trials of the German High-Grade Non-Hodgkin’s Lymphoma Study Group (DSHNHL). Leukemia.

[9] Chapuy B (2018). Molecular subtypes of diffuse large B cell lymphoma are associated with distinct pathogenic mechanisms and outcomes. Nat. Med..

[10] Schmitz R (2018). Genetics and pathogenesis of diffuse large B-cell lymphoma. N. Engl. J. Med..

[11] Reddy A (2017). Genetic and functional drivers of diffuse large B cell lymphoma. Cell.

[12] Akyurek N, Uner A, Benekli M, Barista I (2012). Prognostic significance of MYC, BCL2, and BCL6 rearrangements in patients with diffuse large B-cell lymphoma treated with cyclophosphamide, doxorubicin, vincristine, and prednisone plus rituximab. Cancer.

[13] Johnson NA (2012). Concurrent expression of MYC and BCL2 in diffuse large B-cell lymphoma treated with rituximab plus cyclophosphamide, doxorubicin, vincristine, and prednisone. J. Clin. Oncol..

[14] Rosenwald A (2019). Prognostic significance of MYC rearrangement and translocation partner in diffuse large B-cell lymphoma: a study by the Lunenburg Lymphoma Biomarker Consortium. J. Clin. Oncol..

[15] Swerdlow SH (2016). The 2016 revision of the World Health Organization classification of lymphoid neoplasms. Blood.

[16] Stasik CJ (2010). Increased MYC gene copy number correlates with increased mRNA levels in diffuse large B-cell lymphoma. Haematologica.

[17] Yoon SO (2008). MYC translocation and an increased copy number predict poor prognosis in adult diffuse large B-cell lymphoma (DLBCL), especially in germinal centre-like B cell (GCB) type. Histopathology.

[18] Li S (2015). B-cell lymphomas with concurrent MYC and BCL2 abnormalities other than translocations behave similarly to MYC/BCL2 double-hit lymphomas. Mod. Pathol..

[19] Pophali PA (2020). High level MYC amplification in B-cell lymphomas: is it a marker of aggressive disease?. Blood Cancer J..

[20] Green TM (2012). Immunohistochemical double-hit score is a strong predictor of outcome in patients with diffuse large B-cell lymphoma treated with rituximab plus cyclophosphamide, doxorubicin, vincristine, and prednisone. J. Clin. Oncol..

[21] Quesada AE (2017). Increased MYC copy number is an independent prognostic factor in patients with diffuse large B-cell lymphoma. Mod. Pathol..

[22] Lu T-X (2015). MYC or BCL2 copy number aberration is a strong predictor of outcome in patients with diffuse large B-cell lymphoma. Oncotarget.

[23] Cerhan JR (2017). Cohort profile: the lymphoma specialized program of research excellence (SPORE) molecular epidemiology resource (MER) cohort study. Int. J. Epidemiol..

[24] Lenz G (2008). Stromal gene signatures in large-B-cell lymphomas. N. Engl. J. Med..

[25] Scott DW (2014). Determining cell-of-origin subtypes of diffuse large B-cell lymphoma using gene expression in formalin-fixed paraffin-embedded tissue. Blood.

[26] Hans CP (2004). Confirmation of the molecular classification of diffuse large B-cell lymphoma by immunohistochemistry using a tissue microarray. Blood.

[27] Wang Y (2019). Amplification of 9p24.1 in diffuse large B-cell lymphoma identifies a unique subset of cases that resemble primary mediastinal large B-cell lymphoma. Blood Cancer J..

[28] Team, R. C. *R: A Language and Environment for Statistical Computing*. 3.6.0 ed. (R Foundation for Statistical Computing, 2019).

[29] Kassambara, A., Kosinski, M., & Biecek, P. Survminer: Drawing Survival Curves Using “ggplot2” (2018).

[30] Niitsu N, Okamoto M, Miura I, Hirano M (2009). Clinical features and prognosis of de novo diffuse large B-cell lymphoma with t(14;18) and 8q24/c-MYC translocations. Leukemia.

[31] Swerdlow SH (2014). Diagnosis of ‘double hit’ diffuse large B-cell lymphoma and B-cell lymphoma, unclassifiable, with features intermediate between DLBCL and Burkitt lymphoma: when and how, FISH versus IHC. Hematol. Am. Soc. Hematol. Educ. Progr.

[32] Staiger AM (2017). Clinical impact of the cell-of-origin classification and the MYC/BCL2 dual expresser status in diffuse large B-cell lymphoma treated within prospective clinical trials of the german high-grade non-Hodgkin’s Lymphoma Study Group. J. Clin. Oncol..

[33] Hilton LK (2019). The double-hit signature identifies double-hit diffuse large B-cell lymphoma with genetic events cryptic to FISH. Blood.

