# Supplementary material for: Somatic copy number gains in MYC, BCL2, and BCL6 identifies a subset of aggressive alternative-DH/TH DLBCL patients
Source: Blood Cancer J. 2020 Nov 9;10(11):117. doi: 10.1038/s41408-020-00382-3 (PMC7652824; doi:10.1038/s41408-020-00382-3)
Supplement: Supplementary file 1 — Supplemental Figures [file 41408_2020_382_MOESM1_ESM.pdf]

Supplemental Table 1. Clinial Characteristics by Cohort

Measurement	Mayo Cohort	Chapuy *et al*. Cohort	P
N	260	219	
Age			**< .001**
>60	155 (59.6%)	180 (82.2%)	
<=60	105 (40.4%)	39 (17.8%)	
Sex			.411
Male	151 (58.1%)	119 (54.3%)	
Female	109 (41.9%)	100 (45.7%)	
IPI			.496
0-1	106 (40.8%)	78 (35.6%)	
2	65 (25.0%)	50 (22.8%)	
3	60 (23.1%)	61 (27.9%)	
4-5	29 (11.1%)	28 (12.8%)	
		NA = 2	
Stage			.560
I-II	113 (43.5%)	101 (46.1%)	
III-IV	147 (56.5%)	118 (53.9%)	
Treatment			
R-CHOP	194 (74.6%)	Non-R-CHOP-like	
R2-CHOP	23 (8.8%)	Chemo = 4 (1.8%)	
MR-CHOP	21 (8.1%)		
R-EPOCH	12 (4.6%)		
COO			**< .001**
ABC-like	82 (31.6%)	87 (39.7%)	
GCB	136 (52.3%)	67 (30.6%)	
Unclassified	12 (4.6%)	27 (12.3%)	
NA	30 (11.5%)	38 (17.4%)	
EFS24			.931
Achieved	180 (69.2%)	153 (69.9%)	
	Not reached n=1	Not reached n=13	
Group			**< .001**
DH/TH	14 (5.4%)	7 (3.2%)	
Alternative-DH/TH	24 (9.2%)	26 (11.9%)	
Translocation Positive	93 (35.8%)	55 (25.1%)	
Copy Gain Positive	51 (19.6%)	82 (37.4%)	
No Alteration	78 (30.0%)	49 (22.4%)	

Abbreviations: IPI, International Prognostic Index; COO, Cell-of-Origin; ABC-like, Activated B-cell Like; GCB, Germinal Center B-cell; NA, Not Applicable; EFS24, Event Free Survival at 24 months; DH/TH, Double-Hit/Triple-Hit

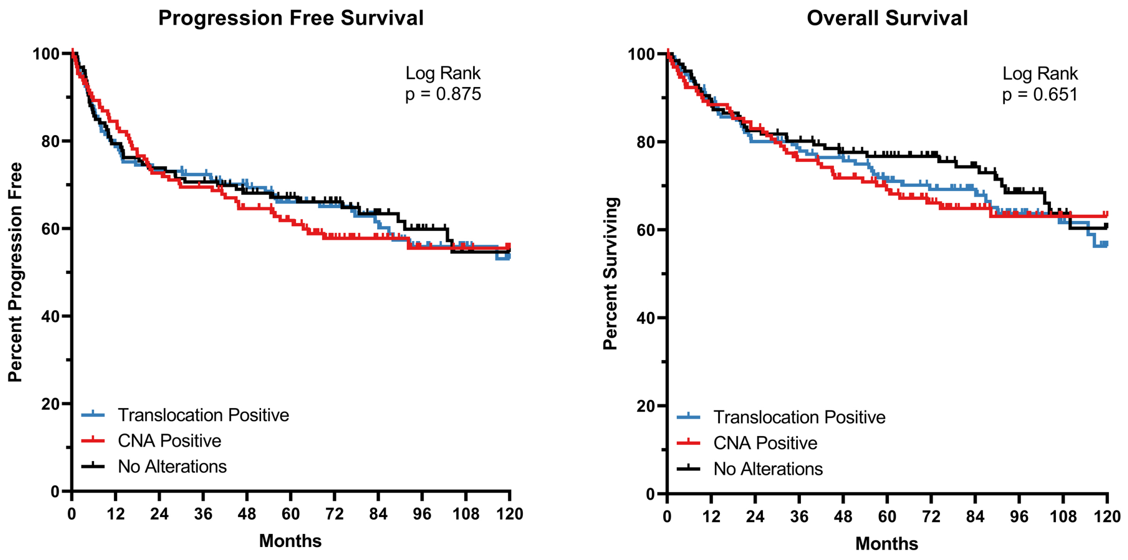

Supplemental Figure 1 - Survival outcomes of control patient groups. Kaplan-Meier curves for Progression Free (EFS/PFS) and Overall Survival of Translocation Positive, CNA Positive, and No Alterations patient groups. P values listed on each graph were derived from a Log Rank test.
